# Dental and Occlusal Changes during Mandibular Advancement Device Therapy in Japanese Patients with Obstructive Sleep Apnea: Four Years Follow-Up

**DOI:** 10.3390/jcm11247539

**Published:** 2022-12-19

**Authors:** Eri Ishida, Ryo Kunimatsu, Cynthia Concepcion Medina, Koji Iwai, Sayumi Miura, Yuji Tsuka, Kotaro Tanimoto

**Affiliations:** Department of Orthodontics and Craniofacial Developmental Biology, Graduate School of Biomedical and Health Sciences, Hiroshima University, Hiroshima 734-8553, Japan

**Keywords:** cephalometry, dental model, long-term treatment, mandibular advancement device, obstructive sleep apnea syndrome, oral appliance, three-dimensional analysis

## Abstract

Dentoskeletal changes caused by the long-term use of mandibular advancement devices (MADs) for obstructive sleep apnea (OSA) have rarely been investigated in Japan. We assessed the long-term dentofacial morphological changes in 15 Japanese patients with OSA who used two-piece MADs for an average of 4 years. Lateral cephalography analyses were performed initially and 4 years later (T1). The dental assessment included overjet, overbite, upper anterior facial height, lower anterior facial height (LAFH), total anterior facial height (TAFH), and anterior facial height ratio. Dental casts were digitized and analyzed using a 3D scanner. Changes in the apnea hypopnea index (AHI) and other sleep-assessment indices were assessed using polysomnography and out-of-center sleep testing. Radiography revealed lingual inclination of the maxillary central incisors, labial inclination of the mandibular central incisors, clockwise rotation of the mandible, and an increase in the TAFH and LAFH at T1. In the dental cast analysis, the diameter width and palatal depth tended to decrease and increase, respectively. There was a significant decrease in the AHI and other sleep assessment indices after using the MADs for approximately 4 years. However, these findings do not provide a strong basis and should be interpreted cautiously. Future studies should have a larger sample size and should further investigate the long-term occlusal and dental changes caused by the original MADs in Japanese patients with OSA.

## 1. Introduction

Obstructive sleep apnea (OSA) is a prevalent and significant public health issue characterized by repeated episodes of upper airway collapse during sleep and is associated with several comorbidities, such as daytime sleepiness, fragmented sleep, and in more severe cases, increased cardiovascular risks [1,2,3,4]. The consensus among the dental community that treats OSA has long been that obesity, as well as certain craniofacial characteristics, carries an increased risk of OSA [5,6].

OSA therapies include conservative treatment (i.e., weight loss, better sleeping postures, alcohol avoidance), upper airway operations, nasal continuous positive airway pressure (nCPAP), and oral appliances (OAs) [7]. nCPAP is considered the gold standard for OSA treatment; however, OAs can be used as an alternative and are often preferred by patients. For mild to moderate OSA, patients are generally instructed to undergo OA therapy [8,9]. Moreover, OAs have also been proposed for apnea patients who are intolerant to CPAP and for patients with snoring with apnea who are unable to incorporate conservative lifestyle changes (i.e., weight loss) [10]. In Japan, OA therapy has become a valid therapy covered by insurance. With this, many patients diagnosed with OSA were referred to dental clinics that provided OA therapy, thus increasing awareness and patient collaboration [11]. Various designs for OAs have been implemented over the years, and most of these have been approved by the US Food and Drug Administration. Generally, the designs have two variants: a tongue-retaining device or a mandibular advancement device (MAD) [12]. An MAD can reduce the apnea–hypopnea index (AHI), oxygen desaturation, and arousal index and increase oxygen saturation, although to a lower extent than nCPAP in patients with OSA [13]. However, the MAD reportedly has equivalent effectiveness compared with nCPAP in daytime sleepiness and hypertension reduction and quality of life (QOL) improvement [13]. In addition, MADs can be useful in primary snoring, as they improve sleep quality and QOL and reduce snoring frequency and intensity [14,15].

Compared to nCPAP therapy, patients reported a preference for MADs despite the effectiveness of the former [16,17,18]. Furthermore, OAs have greater device adherence and less possibility of treatment discontinuation attributed to side effects (odds ratio of discontinuation of treatment attributed to the use of an OA vs. CPAP: 0.54:1) [13].

The mechanical action of these appliances includes repositioning the lower jaw to a more forward position to enlarge the upper airways [14]. This type of appliance can have two designs: an integrated MAD or a two-piece MAD [15].

According to a survey in 2017 by the Japanese Society of Dental Sleep Medicine, approximately 60% of respondents produced an integrated MAD, and the remaining 60% recommended both integrated and two-piece MADs. The continuous use of these appliances has several side effects. These effects vary depending on the short- or long-term use of MADs. Short-term side effects include mouth dryness or hypersalivation, tooth discomfort or oral mucosa swelling, occlusal discomfort when waking up, or temporomandibular joint pain. Long-term side effects include excessive lingual inclination of the mandibular teeth, labial inclination of maxillary teeth, and overjet (OJ) and overbite (OB) reduction [15,19,20]. Unfortunately, there is a lack of information on these changes in the Japanese population.

Our study group has been refining MADs in a segregating design. In a previous case report, the MAD design used in this study was reported to be highly efficient, inexpensive, easy to fabricate, and friendly to intra- and extra-oral tissues [21]. However, no long-term clinical studies have been conducted on the efficacy or side effects of this MAD. Therefore, along with the previously mentioned changes, our study aimed to assess the severity of the long-term changes in Japanese individuals with OSA, including possible skeletal items (such as SNA, SNB, ANB, SN/MP), dental items (such as OB, OJ, U1 to FH, L1 to MP), dentition changes (such as width of dentition), and sleep-breathing assessments (such as AHI) that could occur following the consecutive use of the original two-piece MAD design.

This study was conducted among patients who had undergone MAD therapy for a minimum period of 4 years.

## 2. Materials and Methods

### 2.1. Sample Size Estimation

Sample size calculation was performed using G*Power 3.1 software (Heinrich Heine University, Dusseldorf, Germany) analysis with an error probability of 80% and 0.05. According to the results, a sample size of 35 participants was considered appropriate. We aimed to recruit 40 patients in the group to compensate for any possible dropout.

### 2.2. Participants

**Figure 1:**
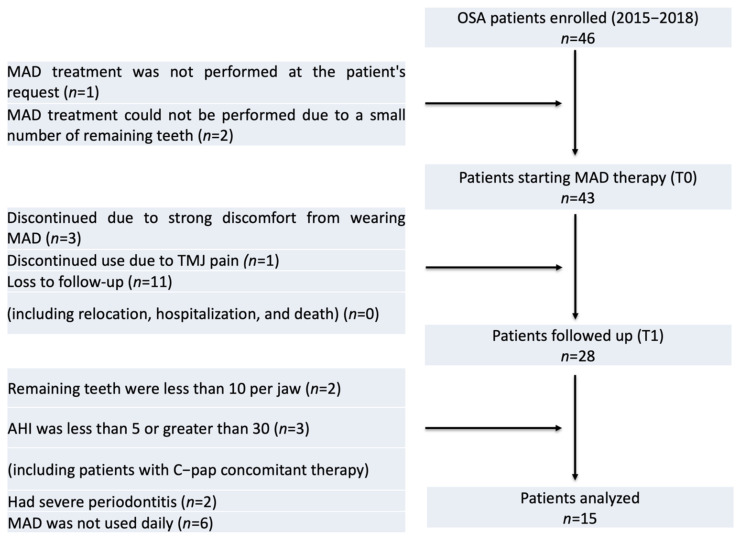
Flowchart illustrating the enrollment and the diagnosis procedure. OSA, obstructive sleep apnea; MAD, mandibular advancement device; TMJ, temporomandibular joint; PSG, polysomnography.

The study was performed among patients with OSA who presented to the Department of Orthodontics, Hiroshima University, between 2015 and 2018. This study was approved by the Ethics Review Committee of Hiroshima University (E-2015-0056). In accordance with the Declaration of Helsinki, the patients provided informed consent to participate in this research. A follow-up flow diagram of the patient selection is shown in Figure 1. The inclusion and exclusion criteria are listed in Table 1. Data were collected at two time points; T0 (initial record) and T1 (4 years and 4 months after the initial visit).

This study included fifteen adults (9 men, 6 women; average age, 64.10 ± 11.90 years old; body mass index, 22.8 ± 3.3) previously diagnosed with mild-to-moderate OSA (apnea–hypopnea index [AHI], 17.73 ± 9.21) by specialized health facilities and referred to the orthodontic department of Hiroshima University Hospital to undergo MAD therapy; patients who met the criteria underwent a 4-year course of MAD therapy.

### 2.3. MAD

**Figure 2:**
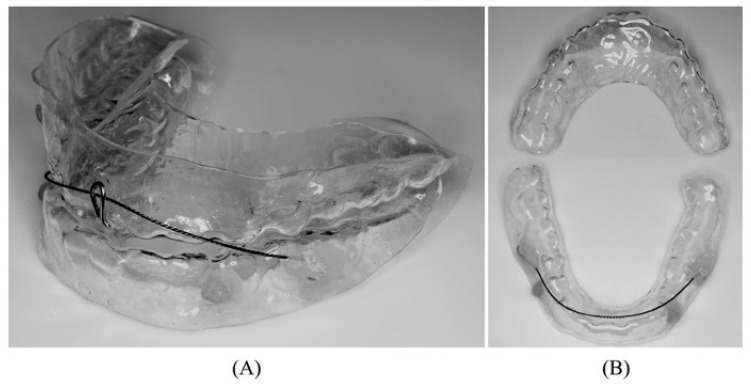
Design of the two-piece MAD used in this study. (**A**) lateral view and (**B**) upper and lower plates. MAD: mandibular advancement device.

The MAD used in this study consisted of two occlusal splints held together by a twisted 0.019-inch orthodontic wire [21]. Two 1.5 mm thick acrylic resin plates (Imprelon, JM Ortho, Tokyo, Japan) were used on each dental arch. These were then molded using a dental pressure molding machine (Biostar VI, JM Ortho, Tokyo, Japan). A 0.019-inch diameter flexible orthodontic wire (MEMOREX, Lancer Vista, USA) was then bonded onto the buccal side of the first permanent molar on the lower splint with an auto-polymerized resin (Orthofast, GC, Tokyo, Japan); however, for the maxillary plate, a 0.8 mm cobalt–chromium alloy wire (Dentsply Sirona, Tokyo, Japan) was bent into a shape of a tear drop hook and bonded with the same resin at the midpoint between the upper central incisors. The hook had an average height of 7 mm. The mandibular position was determined by measuring the maximum mandibular orientation using George Gauge (Johns Dental Laboratories, Terre Haute, IN, USA). The average mandibular advancement was of 4.9 ± 1.7 mm. The length of the lower wire was fixed at two-thirds of the measured maximum advanced position (Figure 2). The forward mandibular position could be adjusted from its initial position by adjusting the length of the twisted lower wire as necessary. With this design, the patient wears both plates and by sliding the mandible forward, the wire is placed on the mandibular splint behind the hook on the maxillary splint, thus fixing the lower jaw in the predetermined forward position, but with freedom to perform lateral movements [13].

### 2.4. Cephalometric Evaluation

**Figure 3:**
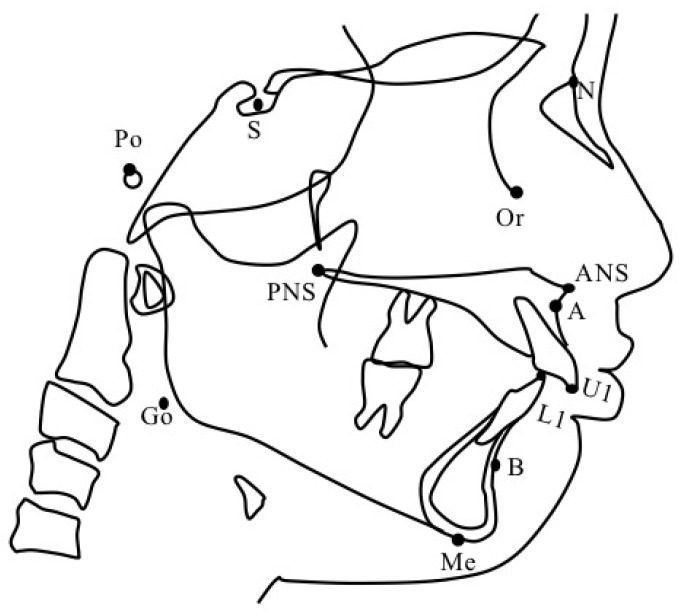
Anatomical landmarks and points of reference. S: Center of sella turcica. N (nasion): Most anterior point of the frontonasal suture. Or (Orbitale): Lowest point on the average left and right inferior borders of the bony orbit. Po (Porion): Highest point on the superior surface of soft tissue of the external auditory meatus. ANS: Apex of the anterior nasal spine. PNS: Intersection between the nasal floor and the posterior contour of the maxilla. A: Most posterior point on the anterior contour of the upper alveolar process. B: Most posterior point on the anterior contour of the lower alveolar process. Me (menton): the lowest point on the symphysis of the mandible. Go (Gonion): Most posterior–inferior point on the convexity of the angle of the mandible. U1: maxillary central incisor. L1: mandibular central incisor.

**Figure 4:**
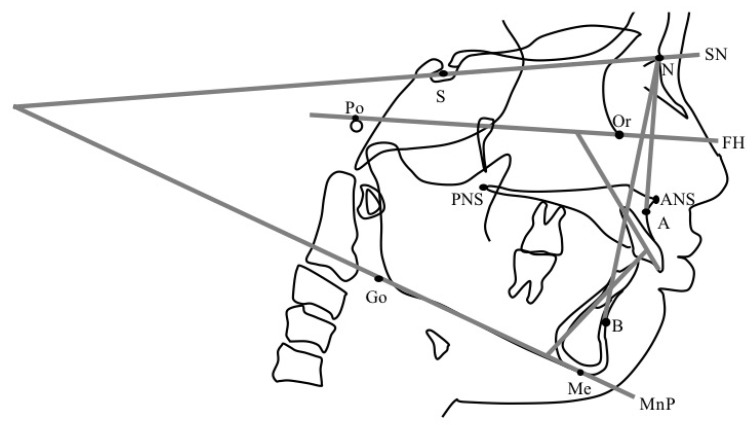
Anatomical angles analyzed in this study. SNA: the angle between the S–N plane and the straight-line N–A. SNB: the angle between the S–N plane and the straight-line N–B. ANB: the angle between the straight-line N–A and N–B. MnP: mandibular plane according to Downs; the plane connecting through Go and Me. FH (Frankfort horizontal) plane: the plane that connects Po and Or. SN/MP: the angle created between the SN plane and the MnP plane. U1 to FH: the angle between the U1′s axis and FH plane. L1 to MnP: the angle between L1′s axis and MnP. Interincisal angle: measures the degree of procumbence of the incisor teeth.

**Figure 5:**
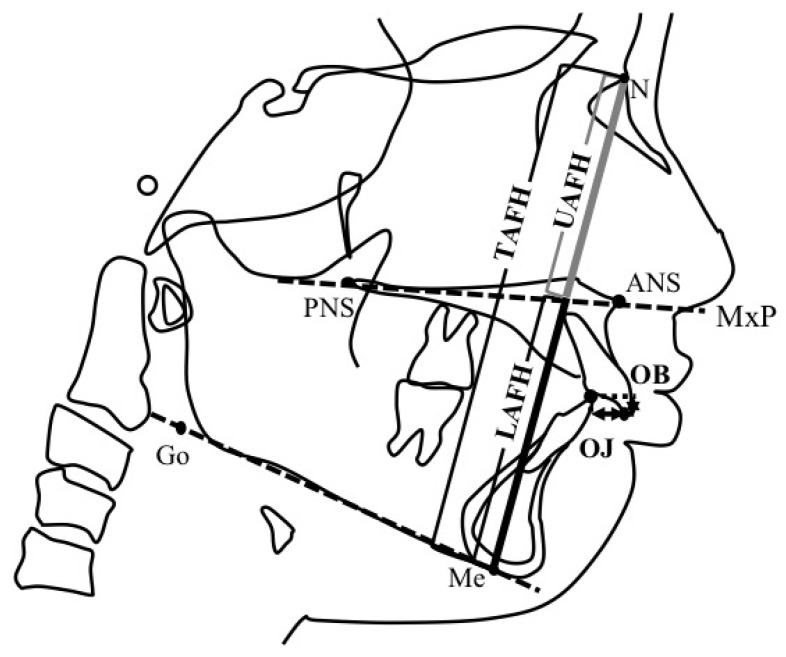
Anatomical planes of reference. N–Me: the plane that connects N and Me. MxP: maxillary plane, the line through ANS and PNS. Overjet (OJ): the horizontal overlap of maxillary incisors over the mandibular incisors (mm). Overbite (OB): the vertical overlap of maxillary incisors over mandibular incisors (mm). UAFH (upper anterior facial height): linear measurement from Nasion to Anterior Nasal Spine (N–ANS). LAFH (Lower anterior facial height): linear measurement from Anterior Nasal Spine to Menton (ANS–Me). TAFH (total anterior facial height): linear measurement from Nasion to Menton (N–Me). Anterior facial height ratio: the ratio calculated between UAFH and LAFH (%).

A series of lateral cephalometric radiographs of the participants was obtained in the upright natural head position with the Frankfort horizontal plane (FH plane) parallel to the floor. Physical changes were assessed at the two time points. The first one was T0 at the initial visit and T1 at 4 years (4y4m ± 1y5m) after the start of MAD therapy. Tracing was performed on each lateral cephalometric radiograph with retracing performed after a month to check for inconsistencies. The anatomical landmarks used in our evaluation are shown in Figure 3. Angular and linear measurements were performed, and anatomical planes were evaluated (Figure 4 and Figure 5). The lateral cephalometric X-ray image extraction and tracing were performed by one examiner, an orthodontist in an academic institution. The examiner analyzed the images at least three months apart. Digitizing and analyzing the measurements were performed using orthodontic analysis software (COA5; JM Ortho, Tokyo, Japan). The upper anterior facial height (UAFH), lower anterior facial height (LAFH), total anterior facial height (TAFH), and anterior facial height ratio were measured using a caliper.

### 2.5. Dental Casts Evaluation

**Figure 6:**
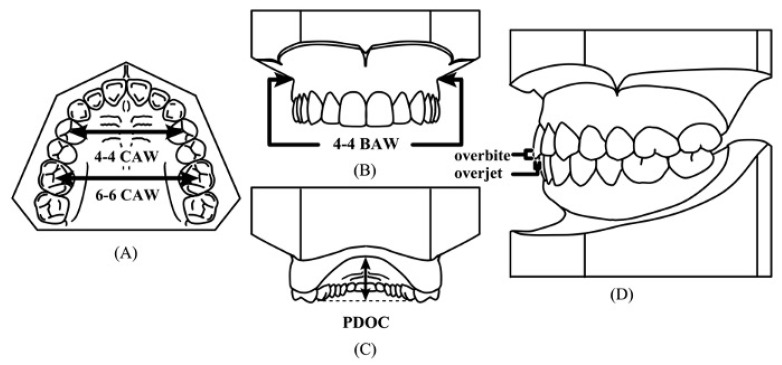
Odontometric variables used in the dental casts analysis. (**A**) Crown arch width (4-4 CAW): measured width of the dental arch between the buccal cusps of the first permanent premolars. Crown arch width (6-6 CAW): measured width of the dental arch between the mesio-buccal cusps of the first permanent molars. (**B**) Basal arch width (4-4 BAW): measured width of the basal arch between the most concave points of the basal bone at the first permanent premolar area. (**C**) Palatal depth from occlusal plane (PDOC): the deepest point of the palate intersecting at a point perpendicular to the mid-palatal raphe and a line connecting the mesio-buccal cusps of the first permanent molars bilaterally. (**D**) Overjet and overbite, as defined in the previous section.

To confirm in a physical and tangible way that patients who undergo MAD therapy indeed experience changes in their dental occlusion, dental casts were obtained and assessed. Both maxillary and mandibular impressions were made with dental alginate at two time points, T0 and T1. Additionally, a wax bite impression in the centric relation was made to obtain the patients’ occlusal records at the same time points. These casts and wax bites were then scanned using a dental 3D scanner (3Shape R700, Ai Technology, Copenhagen, Danmark). The models were first digitized by initially scanning the upper and lower cast separately and then occluding them, using the wax bite to accurately reproduce each patient’s occlusal record. The acquired 3D models were then analyzed using the proprietary analysis module (OrthoAnalyzer^TM^). The measurements evaluated from the 3D scans are presented in Figure 6.

### 2.6. Polysomnography (PSG) and Out-of-Center Sleep Testing (OCST)

All subjects had undergone PSG or OCST at a medical institution prior to initial presentation at our department. The test findings were used to evaluate OSA manifestations at T0. PSG or OCST were performed again at the referring institution at T1, with the patient wearing an MAD, to determine the efficacy of MAD treatment. The respiratory event index (REI) of the participants was assessed. 

### 2.7. Statistical Analysis

A prior sample size was calculated using G*Power 3.1 software (Heinrich Heine University, Dusseldorf, Germany) with the power of the statistical test and the error probability set at 80% and 0.05, respectively. According to the results, a sample size of 35 participants was considered appropriate. After completion of the present study, post hoc analysis was used to calculate the test power using G*Power 3.1 software (Heinrich Heine University, Dusseldorf, Germany) to ascertain the degree of statistical power.

All analyses were performed using the statistical software JMP 15 (SAS Institute Inc., Cary, NC, USA). Data are presented as mean ± standard deviation (SD). Wilcoxon signed-rank test was used to determine the significance of the casts’ assessment. Differences were considered statistically significant at a *p*-value of ≤0.05. The same test was used to compare the differences between the baseline (T0) and follow-up (T1) cephalometric analyses. The intra-examiner intraclass correlation coefficient (ICC) was used to confirm the reliability of the cephalometric evaluation. Values from two sets of cephalometric analyses were used to calculate the intra-examiner ICC. The ICCs of each parameter were calculated using the SPSS software (Statistical Package for Social Sciences version 26.0, Chicago, IL, USA) and/or Microsoft Excel-based software. 

## 3. Results

### 3.1. Cephalometric Analysis

We first compared the intra-examiner ICCs for all parameters with 15 samples together. The intra-examiner ICCs for each parameter are listed in Table 2.

The agreement level was interpreted as suggested by Koo et al. [22]. Values less than 0.5 indicated poor reliability, values between 0.5 and 0.75 indicated moderate reliability, values between 0.75 and 0.9 indicated good reliability, and values greater than 0.90 indicated excellent reliability [23]. Ui-Ui’ levels of the ICC at T0, Li-Li’ levels of the ICC, and Ui-Ui’ levels of the ICC at T1 showed values of 0.694, 0.692, and 0.607, respectively, which were lower than 0.75, so they were moderate reliability. The other values showed high reliability values because they were more than 0.75. The ICC results demonstrated a highly reliable assessment of the measured lateral cephalometric values. After tracing, digitizing, and determining the measurements to be evaluated, certain parameters such as SNA, SNB, and ANB were used to determine the participant’s skeletal pattern, which was found to be mainly skeletal Class II (ANB ≧ 5.96 ± 2.30); however, these values did not change significantly from T0 to T1. Other measurements, such as SN/MP, increased significantly from T0 to T1. Concerning the dental changes as assessed from the lateral cephalograms, the average U1 to FH decreased by an average of 3.21°. Conversely, L1 to MP significantly increased by 1.67 degrees from T0 to T1. These changes were also reflected in the differences in OJ and OB between the same timepoints, with a decrease of 1.07 mm and 0.99 mm, respectively. The interincisal angle increased by 0.86° at the time of evaluation, although no statistically significant difference was found.

The mean UAFH increased from 58.38 ± 3.73 mm to 58.60 ± 3.60 mm in the evaluated time; however, no statistically significant difference was found. The average LAFH and TAFH also significantly increased from 74.18 mm to 74.88 mm and from 132.55 mm to 133.48 mm, respectively. The anterior facial height ratio decreased from 79.05 ± 7.07% to 78.52 ± 6.01%, but the difference was not statistically significant (Table 3). Regarding the post hoc analyses, many variables demonstrated a statistical power of ≥0.8, while the power of ANB˚ was 0.68 and that of the OB was as low as 0.32. For post hoc analyses, all variables showed a statistical power of ≤0.8. OJ showed a moderate statistical power of 0.51, while OB and U1-U1′ had statistical powers of 0.23 and 0.29, respectively. Additionally, the other items showed low statistical power of ≤0.2.

### 3.2. Dental Cast Assessment

After scanning both dental casts, the following changes were observed: even though 4-4 BAW and 6-6 CAW experienced a decreasing trend, no statistically significant difference was found. At T1, the measurement of 4-4 CAW showed a significant decrease compared with at T0. Furthermore, the palatal depth from the occlusal plane (PDOC) showed an increasing trend over time, despite the lack of statistically significant differences. Both OJ and OB significantly decreased at T1. The right and left OJ decreased from an initial 3.58 ± 1.66 and 3.36 ± 1.97 to 2.97 ± 1.45 and 1.99 ± 1.51, respectively, with statistically significant differences found for the left incisor relationship only. The right and left OB significantly decreased from 5.51 ± 2.52 to 3.87 ± 3.12 and 4.93 ± 3.10 to a final 3.69 ±3.36, respectively (Table 4). In the post hoc power analysis, the OJ showed a large power of 0.7–0.8. OB (R1) had a statistical power of 0.51 and OB (L1) had a moderate-to-low statistical power of 0.27. The other items showed a low statistical power of ≤0.2 (Table 4).

### 3.3. AHI, PSG, and OCST

**Figure 7:**
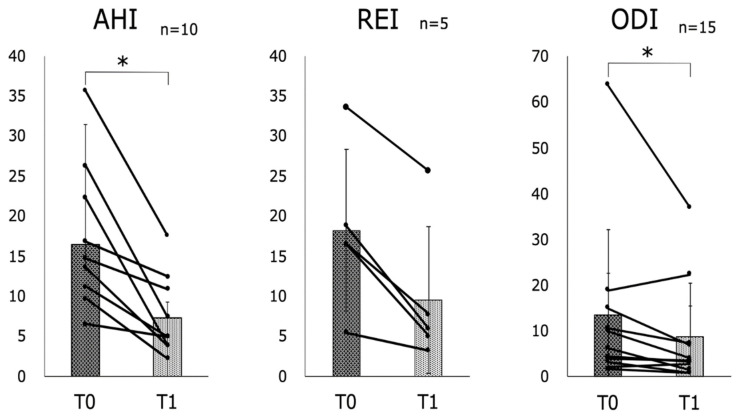
Longitudinal assessment with polysomnography (PSG) and out-of-center sleep testing (OCST). * *p* < 0.01.

Ten patients underwent PSG between T0 and T1, and five patients underwent OCST. The mean AHI based on PSG was 16.5 at T0 and 7.2 at T1, with a significant reduction (*p* < 0.01). Similarly, the mean ODI of the 15 patients who underwent PSG or OCST was 13.5 at T0 and 8.6 at T1, showing a significant reduction (*p* < 0.01). The mean REI based on OCST was 18.2 for T0 and 9.5 for T1, with a tendency for reduction, although no significant differences were found (Figure 7).

In the post hoc power analyses, AHI resulted in a power of ≥0.8, while REI and ODI had lower powers of 0.31 and 0.18, respectively (Table 5).

Each patient’s respective information (age, sex, molar relationship, maxillofacial skeletal pattern, body mass index (BMI), pre- and post-treatment evaluation, and anterior movement of the mandible) is presented in Table 6.

There was a significant reduction in the mean AHI of the 10 subjects who underwent PSG testing for sleeping about 4 years after using the isolated MAD (*p* < 0.01). In contrast, no significant reduction was observed in the mean REI of the five individuals who underwent OCST. There was also a significant reduction in the mean ODI of the 15 individuals who underwent PSG or OCST (*p* < 0.01). The black line in the diagram shows the change in T0 and T1 per patient. AHI: apnea–hypopnea index; REI: respiratory event index; ODI: oxygen desaturation index; MAD: mandibular advancement device; PSG: polysomnography; OCST: out-of-center sleep. * *p* < 0.01.

## 4. Discussion

### 4.1. Cephalometric Analysis

The short-term continuous use of an MAD has been reported to lead to temporomandibular joint discomfort upon waking up, excessive salivation, intraoral and lip dryness, and improper tooth contact upon removing the MAD [23,24,25]. However, patients find that these symptoms improve throughout the day, and the level of discomfort is reportedly minimal [17,18,26,27,28,29,30]. On the contrary, long-term dental changes or changes in occlusion are harder for the patients to notice [16]; these include OJ and OB reduction, a forward shift of the mandibular molars, and increased maximal opening [24,25,26,31,32,33,34]. The findings in this study demonstrate that, as previously shown in other studies with other ethnicities, Japanese OSA patients also experience several dentoskeletal changes after a continuous long-term use of MAD therapy.

The average skeletal patterns remained stable, although a small increase in SNA and a small decrease in SNB angles were observed; these findings were not significant, and thus clinically negligible. Intriguingly, the ANB value increased from an initial value of 5.96 ± 2.30° at T0 to 6.15 ± 2.16° at T1, although no statistical significance was found. This increase was consistent with the results obtained by Robertson et al. [30]. However, this ANB value should be cautiously interpreted, as its statistical power in the post hoc analyses was as low as 0.05. In the future, sufficient verification by increasing the number of samples is necessary.

Regarding other skeletal measurements, such as SN/MP, which measure the degree of mandibular opening related to the cranial vault, showed an increase. This clockwise rotation of the mandible upon mandibular opening can be explained as a counteract effect to the forward movement enforced by the MAD. Similar to previous findings [24,25,26,31,32,33,34], OJ and OB were significantly reduced by 1.07 mm and 0.99 mm, respectively. These results can be attributed to the retroclination of the upper incisors and proclination of the lower incisors. This can be confirmed while assessing the incisal changes in the lateral cephalogram, as expressed by U1 to FH and L1 to MnP measurements. The average observed retroclination of the upper incisors was 1.67°, and the average proclination of the lower incisors was 3.2°. These changes from T0 to T1 can be explained as when the MAD is being used, a labially directed force and lingually directed force actively push both lower and upper incisors, respectively. A direct consequence of changes in the incisal inclination is an increase in the interincisal angle, although no statistical significance was found. These results agree with those obtained by Almeida et al. [14], which were obtained using the Klearway appliance (Klearway, Vancouver, British Columbia, Canada). Furthermore, these dental changes are more closely related to the movement of the mandible brought upon by the MAD, rather than its design or material. Several studies have reached this conclusion [14,30,35,36].

With regard to vertical changes, the UAFH remained stable and unchanged during the evaluation period. However, the LAFH significantly decreased at T1. In contrast, the TAFH significantly increased over the same period, with an average increase of 0.93 mm. This could be correlated and attributed to an increase in the SN/MP value over time, which is also caused by the previously explained clockwise mandibular rotation due to long-term MAD use. Since these results were derived following a 4-year continuous wear of an MAD, it can be inferred that the longer a patient wears such devices, the greater the increase in vertical measurements is. These results are similar to those obtained by Wang et al. [36], where the same conclusions were reached regarding the TAFH measurements. However, this increase has also been reported in studies with short-term evaluations [37]. Furthermore, the increase in the LAFH corresponds to and correlates with the increase in OB observed in this study.

However, all items in the cephalometric analysis had a power of 0.8 or less in the post hoc analysis. Notably, the changes in the cephalometric analysis should be interpreted with caution, as many variables had a low statistical power of 0.2 or less. In the future, it is necessary to increase the number of samples for sufficient verification.

### 4.2. Dental Cast Assessment

Plaster dental models have been traditionally used in dentistry to record patients’ diagnosis, treatment planning, and progress in different dental specialties. Especially in orthodontics, it is imperative to preserve these models for medical record keeping. However, storing plaster dental casts may take up a lot of space in private practice; therefore, with the need to reduce space usage and modernize current practices, digital dental models have become a more widely recommended practice [38,39,40]. When comparing traditional plaster models and their digitized counterparts on measurability and reliability, numerous studies have found that the differences between them are negligible [41,42,43,44,45]. Three-dimensional dental models present several advantages to their plaster counterparts, including the ease of obtaining reliable measurements more rapidly [46,47,48]. In this study, the crown and basal arch width measurements showed a decreasing trend over time, although this was not statistically significant. This decrease in width may occur due to the age increase in the subjects, which in turn accelerates bone density decrease and tissue deterioration. An increase of 0.29 mm in PDOC was found during the four-year evaluation. This might be due to the combined pressure of both the MAD and tongue placed on the palatal vault, as well as the deterioration that naturally occurs with aging. OJ and OB measurements on each left and right central incisor provided a more accurate assessment. The results obtained confirmed those from cephalometric analysis. Each individual tooth showed a significant decrease in both the measurements. On average, OJ was significantly reduced on the right and left incisor by 1.41 mm and 1.85 mm, respectively. Additionally, OB was significantly reduced by an average of 1.64 mm and 1.57 mm on the right and left incisor, respectively. These results agree with those obtained by Chen et al. [49]. Overall, it was concluded that the sagittal tooth relationship between the maxillary and mandibular anterior teeth saw a reduction with the continuous use of MADs, irrespective of the design and materials used [18,24,25,31,34,49,50]. In recent systematic reviews, MADs for OSA may reportedly tilt the mandibular incisors to 1.54 ± 0.16˚ labially, reduce the OJ by 0.89 ± 0.04 mm, reduce the OB by 0.68 ± 0.04 mm, and rotate the mandible downward [13]. Similar results were also demonstrated in the present study. However, although the statistical power of overjet on post hoc analysis ranged from 0.8 to 0.7, the outcome should be carefully interpreted because 4-4 CAW, 6-6 CAW, 4-4 BAW, PDOC, and OB showed poor statistical power on post hoc analyses.

### 4.3. AHI, PSG, and OCST

Regarding longitudinal assessment with PSG and OCST, a systematic review of treatment efficacy with an MAD reports a reduction in the AHI from approximately 80% at pretreatment to 21% post-treatment [51]. Age, BMI, anterior translation, and body posture are associated with differences in the treatment efficacy of MADs [52]. In the present study, AHI was reduced by 44% at 4 years after treatment initiation. In addition, there was a 52% reduction in the REI compared with the pretreatment level. Literature on AHI in cases of long-term therapeutic intervention with MADs is rare, in part due to difficulties in unifying the conditions. The present study identified significant reductions in the AHI and ODI at the 4-year follow-up of using isolated MADs. The effectiveness of isolated MADs was demonstrated. The post hoc power was lower for REI and ODI than that for small samples and larger SD. Consequently, the interpretation of REI and ODI values should be conducted with caution, and there is a need to have sufficient samples for validation.

### 4.4. Limitations

This study has several limitations. First, the small number of samples is a key limitation. Due to insufficient sample size, post hoc analyses revealed that most parameters were not adequately powered; they had at least 0.8 of statistical power. Therefore, the results of the present study should be cautiously interpreted. In the future, we need to increase the sample size and further investigate the long-term occlusal and dental changes caused by the original MAD in the Japanese patients with OSA. For the sleeping examination after long-term treatment, both PSG examination and OCST were used. It is considered that accurate outcomes were obtained by collaborating with the medical department and the unification of the PSG examination. Additionally, it would be interesting to assess these changes in a similar period by comparing patients wearing MADs with patients wearing an nCPAP. Since the follow-up period for this study was 4 years, it can be hypothesized that side effects may increase if the evaluation time is longer. In addition, by comparing with the findings of a longer evaluation time, it may be possible to understand the years of MAD use required for side effects to occur. This could be a topic for future studies. We also conducted this study in a single group only with no comparison group to verify the long-term efficacy and side effects of the original MAD. Owing to ethical issues, it was not possible to have a control group that received no intervention; however, the effectiveness and side effects of fixed-bearing MADs versus isolated-bearing MADs need to be verified in the future. In addition, randomized clinical trials providing high-quality evidence are warranted.

Recently, there has been renewed interest in the examination of airways and craniofacial morphology using cone beam computed tomography (CBCT). The present study included two-dimensional cephalography, which is a classic study, because the effect of exposure dose was examined. In a future study, the use of CBCT to evaluate airways and craniofacial morphology should be considered.

## 5. Conclusions

The original MAD used in this study has been shown to improve AHI levels after long-term use for 4 years. Changes in several dental parameters, as well as some skeletal parameters, can occur in individuals who continuously wear MADs for OSA therapy. 

However, the present study results do not provide a strong basis and should be interpreted with caution. In future research, a larger sample size should be used, and there is a need to further investigate the long-term occlusal and dental changes caused by the original MAD in Japanese patients with OSA. 

## Figures and Tables

**Table 1:** Inclusion and exclusion criteria of study participants.

	Inclusion Criteria	Exclusion Criteria
•	Patients diagnosed with mild to moderate OSA with full PSG test result of 5 ≤ AHI < 30	Complications such as serious systemic illnesses (including mental illness) other than OSA
•	Patients with 10 or more existing teeth in both the maxilla and Mandible	Temporomandibular joint symptoms
•	Patients with good periodontal tissue health	Patients with three or less existing molars
•	Patients with no jaw dysfunction	Patients who are continuing dental treatment such as periodontal therapy

AHI, apnea–hypopnea index; OSA, obstructive sleep apnea; PSG, polysomnography.

**Table 2:** The intra-examiner ICCs for each parameter.

Variable	T0			T1		
	ICC	95% CI		ICC	95% CI	
		Lower Bound	Upper Bound		Lower Bound	Upper Bound
**SNA**	0.794	0.081	0.976	0.796	0.089	0.976
**SNB**	0.837	0.207	0.981	0.821	0.159	0.979
**ANB**	0.912	0.491	0.990	0.872	0.328	0.985
**SN/MP**	0.976	0.838	0.997	0.904	0.597	0.993
**U1-FH**	0.949	0.676	0.994	0.994	0.956	0.999
**∠L1 to MP**	0.931	0.584	0.992	0.948	0.670	0.994
**interincisal angle**	0.905	0.461	0.989	0.956	0.714	0.995
**Overjet**	0.954	0.703	0.995	0.946	0.659	0.994
**Overbite**	0.834	0.198	0.981	0.977	0.844	0.998
**Ui-Ui’**	0.820	0.155	0.979	0.756	-0.140	0.971
**Li-Li’**	0.849	0.248	0.983	0.945	0.653	0.994
**U6-U6′**	0.892	0.408	0.988	0.891	0.402	0.988
**L6-L6′**	0.910	0.483	0.990	0.888	0.392	0.987
**UAFH**	0.917	0.515	0.991	0.979	0.855	0.998
**LAFH**	0.994	0.954	0.999	0.989	0.923	0.999
**TAFH**	0.984	0.889	0.998	0.995	0.960	0.999
**Anterior facial height ratio (%)**	0.936	0.610	0.993	0.958	0.729	0.995

ICC: intraclass correlation coefficient; CI: confidence interval; UAFH (upper anterior facial height): linear measurement from nasion to anterior nasal spine (N–ANS); LAFH (lower anterior facial height): linear measurement from anterior nasal spine to menton (ANS–Me); TAFH (total anterior facial height): linear measurement from nasion to menton (N–Me); Anterior facial height ratio: the ratio calculated between UAFH and LAFH (%).

**Table 3:** Changes in the cephalometric values before and after using the MAD.

Variable	T0		T1		*p* Value	Signifcance of the Difference	Effect Size dz	Statistical Power
	Average	S.D.	Average	S.D.				
**SNA (°)**	81.81	2.57	81.90	2.73	0.244		0.0339159	0.0516345
**SNB (°)**	75.85	3.41	75.75	3.43	0.583		0.0292394	0.0512145
**ANB (°)**	5.96	2.30	6.15	2.16	0.310		0.0850761	0.0603352
**SN/MP (°)**	36.07	8.31	36.74	8.45	0.003	**	0.0799439	0.0591198
**U1 to FH (°)**	106.41	8.49	103.21	9.31	0.008	**	0.3584114	0.2425793
**L1 to MP (°)**	98.61	7.36	100.28	7.95	0.030	*	0.2176737	0.1194130
**interincisal** **angle (°)**	127.43	16.37	128.29	16.81	0.277		0.0518248	0.0538212
**overjet (mm)**	4.69	1.95	3.62	1.82	0.003	**	0.5666295	0.5116650
**overbite (mm)**	4.01	2.78	3.02	2.87	0.035	*	0.3503092	0.2338615
**U1-U1′ (mm)**	26.37	2.34	27.34	2.44	0.007	**	0.4055916	0.2967969
**L1-L1′ (mm)**	22.70	3.15	21.76	3.20	0.026	*	0.2960355	0.1804072
**U6-U6′ (mm)**	25.80	2.62	26.23	2.95	0.119		0.1535920	0.0840846
**L6-L6′ (mm)**	17.00	3.21	16.84	2.79	0.413		0.0529456	0.0539886
**UAFH (mm)**	58.38	3.73	58.60	3.60	0.349		0.0599990	0.0551255
**LAFH (mm)**	74.18	5.72	74.88	5.57	0.021	*	0.1239707	0.0720797
**TAFH (mm)**	132.55	7.53	133.48	7.76	0.002	**	0.1216069	0.0712368
**Anterior facial**	79.05	7.07	78.52	6.01	0.229		0.0802530	0.0591908
**height ratio (%)**								

MAD: mandibular advancement device; SD: standard deviation; UAFH (upper anterior facial height): linear measurement from nasion to anterior nasal spine (N–ANS); LAFH (lower anterior facial height): linear measurement from anterior nasal spine to menton (ANS–Me); TAFH (total anterior facial height): linear measurement from nasion to menton (N–Me); Anterior facial height ratio: the ratio calculated between UAFH and LAFH (%). * *p* < 0.05 ** *p* < 0.01

**Table 4:** Changes in the oral model values before and after using the MAD.

Variable	T0		T1		*p* Value	Significance of the Difference	Effect Size dz	Statistical Power
	Average	S.D.	Average	S.D.				
**4-4 CAW (mm)**	42.28	2.68	41.64	1.79	0.039	*	0.2707081	0.1585536
**6-6 CAW (mm)**	46.52	2.90	46.14	2.64	0.531		0.1367331	0.0769231
**4-4 BAW (mm)**	43.01	3.24	42.29	2.39	0.813		0.2474519	0.1402805
**PDOC (mm)**	17.94	2.21	18.23	2.81	0.844		0.1131389	0.0683554
**OJ (R1) (mm)**	3.58	1.66	2.17	1.45	0.195		0.9006138	0.8838789
**OJ (L1) (mm)**	3.36	1.97	1.99	1.51	0.039	*	0.7674980	0.7675206
**OB (R1) (mm)**	5.51	2.52	3.87	3.12	0.016	*	0.5719322	0.5190546
**OB (L1) (mm)**	4.93	3.10	3.69	3.36	0.008	**	0.3829715	0.2700947

MAD: mandibular advancement device; SD: standard deviation; 4-4 CAW (crown arch width): measured width of the dental arch between the buccal cusps of the first permanent premolars; 6-6 CAW (crown arch width): measured width of the dental arch between the mesio-buccal cusps of the first permanent molars; 4-4 BAW (basal arch width): measured width of the basal arch between the most concave points of the basal bone at the first permanent premolar area; OJ (overjet): the horizontal overlap of maxillary incisors over the mandibular incisors (mm); OB (overbite): the vertical overlap of maxillary incisors over mandibular incisors (mm); R1: right central incisor; L1: left central incisor.* *p* < 0.05 ** *p* < 0.01.

**Table 5:** Changes in AHI, REI, and ODI values before and after using the MAD.

Variable	T0		T1		*p* Value	Significance of the Difference	Effect Size dz	Statistical Power
	Average	S.D.	Average	S.D.				
AHI (n = 10)	16.5	9.0	7.2	5.4	0.006	**	1.1853150	0.8978838
REI (n = 5)	18.2	10.1	9.5	9.2	0.125		0.8986280	0.3174512
ODI (n = 15)	13.5	18.5	8.6	11.8	0.002	**	0.3020379	0.1858808

SD: standard deviation; MAD: mandibular advancement device; AHI: apnea–hypopnea index; REI: respiratory event index; ODI: oxygen desaturation index. ** *p* < 0.01.

**Table 6:** Each patient’s respective information.

Subject	Age	Sex	Overjet	Overbite	Skeletal Class	Angle Class		BMI	First (T0)			Follow-up (T1)			Mandibular Advancement	
			(mm)	(mm)		R	L		AHI	REI	ODI	AHI	REI	ODI	(mm)	(%)
1	51Y 3M	F	+3.8	+3.1	Ⅱ	Ⅱ	Ⅱ	31.0	22.3	-	17.4	3.9	-	3.6	5.6	44.6
2	66Y 10M	M	+7.8	+12.1	Ⅰ	Ⅱ	Ⅱ	23.1	16.8	-	2.0	8.5	-	2.7	6.5	80.3
3	40Y 4M	M	+5.0	+5.7	Ⅰ	Ⅱ	Ⅱ	21.3	-	18.8	14.0	-	3.2	7.3	3.8	59.4
4	45Y 1M	F	+4.0	+2.4	Ⅱ	Ⅱ	Ⅱ	21.4	13.6	-	6.2	1.0	-	1.5	5.1	82.1
5	60Y 11M	F	+8.7	+6.3	Ⅱ	Ⅱ	Ⅱ	21.8	13.7	-	10.0	3.8	-	4.1	3.2	30.1
6	78Y 2M	M	+2.2	+1.7	Ⅰ	Ⅰ	Ⅰ	24.6	-	16.5	19.3	-	7.7	14.9	3.4	29.6
7	46Y 2M	M	+3.1	+3.5	Ⅰ	Ⅱ	Ⅰ	18.9	11.2	-	3.2	4.9	-	0.9	4.1	37.8
8	60Y 11M	M	+2.7	+1.2	Ⅱ	Ⅰ	Ⅰ	28.5	46.3	-	63.7	17.5	-	36.9	3.7	64.8
9	42Y 4M	F	+6.9	+5.8	Ⅱ	Ⅰ	Ⅲ	18.0	9.7	-	13.0	2.2	-	9.6	9.3	67.4
10	70Y 10M	M	+3.7	+1.4	Ⅱ	Ⅱ	Ⅰ	22.1	26.3	-	1.6	7.4	-	0.8	5.2	63.2
11	73Y 4M	M	+4.1	+4.9	Ⅰ	Ⅰ	Ⅰ	22.9	-	18.8	12.5	-	5.9	7.5	3.4	53.6
12	61Y 8M	M	+2.4	+1.9	Ⅱ	Ⅰ	Ⅰ	22.1	-	20.3	18.8	-	25.4	22.9	3.5	41.5
13	74Y 3M	M	+5.5	+4.6	Ⅱ	Ⅱ	Ⅱ	22.8	-	16.5	6.7	-	5.0	3.8	7.4	71.0
14	67Y 7M	F	+4.3	+2.9	Ⅱ	Ⅱ	Ⅱ	20.5	6.5	-	4.3	4.9	-	3.2	3.9	49.4
15	62Y 9M	F	+6.1	+2.8	Ⅱ	Ⅱ	Ⅱ	22.7	8.7	-	10.5	14.7	-	7.2	5.0	47.7

BMI: body mass index; AHI: apnea–hypopnea index; REI: respiratory event index; ODI: oxygen desaturation index.

## Data Availability

Not applicable.
